# The Effects of Propofol on Local Field Potential Spectra, Action Potential Firing Rate, and Their Temporal Relationship in Humans and Felines

**DOI:** 10.3389/fnhum.2013.00136

**Published:** 2013-04-09

**Authors:** Sara J. Hanrahan, Bradley Greger, Rebecca A. Parker, Takahiro Ogura, Shinju Obara, Talmage D. Egan, Paul A. House

**Affiliations:** ^1^Departments of Bioengineering, University of UtahSalt Lake City, UT, USA; ^2^Department of Neurosciences, University of UtahSalt Lake City, UT, USA; ^3^Department of Anesthesiology, National Defense Medical CollegeTokorozawa, Japan; ^4^Department of Anesthesiology, Fukushima Medical UniversityFukushima, Japan; ^5^Department of Anesthesiology, University of UtahSalt Lake City, UT, USA; ^6^Department of Neurosurgery, University of UtahSalt Lake City, UT, USA

**Keywords:** microelectrode array, consciousness, power spectrum, propofol, local field potential

## Abstract

Propofol is an intravenous sedative hypnotic, which, acting as a GABA_A_ agonist, results in neocortical inhibition. While propofol has been well studied at the molecular and clinical level, less is known about the effects of propofol at the level of individual neurons and local neocortical networks. We used Utah Electrode Arrays (UEAs) to investigate the effects of propofol anesthesia on action potentials (APs) and local field potentials (LFPs). UEAs were implanted into the neocortex of two humans and three felines. The two human patients and one feline received propofol by bolus injection, while the other two felines received target-controlled infusions. We examined the changes in LFP power spectra and AP firing at different levels of anesthesia. Increased propofol concentration correlated with decreased high-frequency power in LFP spectra and decreased AP firing rates, and the generation of large-amplitude spike-like LFP activity; however, the temporal relationship between APs and LFPs remained relatively consistent at all levels of propofol. The probability that an AP would fire at this local minimum of the LFP increased with propofol administration. The propofol-induced suppression of neocortical network activity allowed LFPs to be dominated by low-frequency spike-like activity, and correlated with sedation and unconsciousness. As the low-frequency spike-like activity increased and the AP–LFP relationship became more predictable firing rate encoding capacity is impaired. This suggests a mechanism for decreased information processing in the neocortex that accounts for propofol-induced unconsciousness.

## Introduction

Propofol (2,6-di-isopropylphenol) is an intravenous sedative hypnotic and is an allosteric modulator of GABA_A_ chloride channels. GABA_A_ chloride channels are more densely located in the neocortex than in subcortical structures, and propofol therefore inhibits neural activity preferentially in the neocortex (Bai et al., 1999; Kaisti et al., 2002; Hentschke et al., 2005; Solt and Forman, 2007). While the pharmacology and electroencephalography (EEG) effects of propofol have been extensively studied, its effects on action potentials (APs) and local field potentials (LFPs) are not yet well understood.

Most studies focusing on the electrophysiological effects of propofol have used low spatial resolution neural recording techniques, such as EEG and electrocorticography (ECoG). EEG investigations demonstrate that increasing propofol concentration in human subjects shifts cortical activity from a high-frequency, low-amplitude signal to a low-frequency, high-amplitude signal. Specifically, with increasing levels of propofol anesthesia, beta activity (13–30 Hz) decreases and alpha (8–12 Hz) and delta activities (0–4 Hz) increase (Feshchenko et al., 2004). As propofol concentration is further increased, the EEG signal develops a burst suppression pattern with flat low-amplitude periods interspaced between high alpha and beta activity (Clark and Rosner, 1973). At the highest levels of propofol sedation, the flat low-amplitude periods lengthen and the EEG signal becomes isoelectric (Claassen et al., 2002). Similar patterns have been seen by using intracranial ECoG recordings in human subjects during induction and emergence from propofol anesthesia. With the induction of anesthesia, delta band (1–2 Hz) power increases as gamma band (37–205 Hz) power decreases. In addition, the correlation between the phase of the delta band (1–4 Hz) and the power of the gamma band (23–165 Hz) strengthens with propofol anesthesia (Breshears et al., 2010).

Power in the high-frequency component of EEG signals corresponds to corticocortical activity (Gray and McCormick, 1996), while power in the low-frequency component of EEG signals primarily arises from subcortical interactions (McCormick and Bal, 1997). The decrease in high-frequency power with the administration of propofol may be due to a decrease in intracortical and corticocortical activity. The increase in low-frequency power may arise from interactions with subcortical structures such as the thalamus (Velly et al., 2007; Ching et al., 2010). Furthermore, the burst suppression pattern observed in the EEG signal in the anesthetized state is comparable with neural signal patterns seen in subcortical structures (Steriade et al., 1994). The activity in these subcortical structures may be observed in the neural signals throughout the cortex in the anesthetized state, resulting in an increased synchronization across the cortex (Steriade et al., 2001).

In contrast to EEG and ECoG that integrate neural signals from large areas of cortex and are spatiotemporally smoothed, LFPs are recorded using high-impedance micro-scale electrodes and are thought to be generated by synaptic potentials (Mitzdorf, 1985; Nunez and Srinivasan, 2006; Katzner et al., 2009; Khawaja et al., 2009). While the spatial extent of LFPs is debated, they likely represent coordinated neural activity of cortical micro-circuits, e.g., cortical columns. EEG and ECoG are unable to record APs from individual neurons or LFPs and are therefore unable to examine AP–LFP relationships (Buzsaki et al., 2012).

Arrays of microelectrodes can be used to examine the changes in LFP and AP activity during anesthesia. Utah Electrode Array (UEA) recordings in the rat cortex during the administration of urethane anesthesia demonstrated an overall decrease in AP firing and an increase in synchronous bursts of AP firing (Erchova et al., 2002). In addition, UEA recordings in the human cortex during the administration of propofol demonstrated a coupling between the slow (<1 Hz) oscillation in the LFP and AP firing (Lewis et al., 2012). In this investigation, we examined the effects of propofol anesthesia on APs and LFPs and on the temporal relationship between these two neural signals. Two human patients and one feline were given propofol by bolus injection. A pharmacokinetic model was developed and was used for the target-controlled continuous infusion of propofol in two additional felines. Given that propofol reduces power in high frequencies and increases power in low frequencies in EEG and ECoG recordings, we hypothesized that similar effects would be seen on microelectrode recordings of APs and LFPs. Also, the effect of propofol on the temporal relationship between APs and LFPs is unknown, and we aimed to determine whether propofol altered this relationship such that information processing in the neocortex would be suppressed.

## Materials and Methods

We used penetrating UEAs to investigate the electrophysiological changes resulting from propofol anesthesia at the level of single neuronal APs and LFPs. Each UEA (Blackrock Microsystems, Salt Lake City, UT, USA) consisted of 96 electrodes, 1 mm in length, spaced 400 μm apart. UEAs were implanted into the cortices of two human patients and three felines. Reference electrodes for each UEA were placed in the subdural space greater than 2 cm away from the UEA. Neural activity was recorded and sampled at 30 kHz using a Cerberus system (Blackrock Microsystems). APs were identified using an automatic spike sorting algorithm (Shoham et al., 2003). The most prominent unit was used for each channel for consistency across all analysis. Neural activity was recorded at different sedation levels.

The study began with two humans (Patients A and B) given boluses of propofol. To evaluate whether the neurophysiological changes seen in the human could be replicated in an animal model under more controlled conditions, one feline (Feline A) was given boluses of propofol. To provide a controlled stepwise administration of propofol, a pharmacokinetic model was developed using a target-controlled infusion (TCI) system for two additional felines (Felines B and C).

### Human studies

The two patients were enrolled in an Institutional Review Board-approved study. Each patient suffered from medically refractory temporal lobe epilepsy; on the basis of a multidisciplinary review process, their condition was presumed to originate from the mesial temporal structures. We evaluated seizure semiology, routine EEG, video EEG, high-resolution MRI, and PET imaging.

In each case, a UEA was implanted in the middle temporal gyrus, approximately 3 cm from the temporal pole. The patients were maintained on a total intravenous anesthetic consisting of propofol and remifentanil during their craniotomy. Once the UEA was implanted, the anesthetic infusions were modulated to obtain a bispectral index scale (BIS) from a BIS Vista Monitoring System (Aspect Medical Systems, Norwood, MA, USA) of ∼50. The UEA made continuous recordings while boluses of propofol were administered intravenously to obtain a BIS of 20–30. The stable anesthetized state achieved before the boluses of propofol were delivered was considered baseline. For both patients, two rounds of BIS suppression with propofol boluses were recorded. The UEA was removed, and a standard anterior temporal lobectomy was then performed on each patient. Patient A (31-year-old man) underwent a right-sided resection, and Patient B (64-year-old man) underwent a left-sided resection. The hippocampus was histologically normal in each case. Specimens of lateral temporal neocortex from each patient contained thickening of the subpial plate, consistent with Chaslin’s marginal sclerosis.

### Feline studies

In an Institutional Animal Care and Use Committee approved study, UEAs were implanted in the motor cortex of three felines. For Feline A, the neural activity was recorded continuously beginning while the feline was fully awake and continuing while it was given two boluses of propofol sufficient to mimic clinical burst suppression patterns in the LFP.

For felines B and C, a TCI system was developed to enable the achievement of pseudo steady-state propofol concentrations at or near a specified plasma level (Egan, 2003). In brief, using raw propofol concentration versus time data obtained in a prior investigation (Bester, 2009), the parameters for a three-compartment mamillary model were estimated using non-linear regression techniques. This feline propofol pharmacokinetic model was then incorporated into Stanpump (Stanford University, Palo Alto, CA, USA), a TCI software package that allows implementation of user-designated models. The TCI system was used to achieve and maintain predicted plasma propofol concentrations that were gradually raised over time. With the pharmacokinetic aspects of the experiment controlled in this manner, the pharmacodynamic measurements (i.e., electrophysiology and clinical observations) were made at each pseudo steady-state concentration plateau.

For Feline B, isoflurane was administered at the beginning of the experiment to place the intravenous catheter for propofol infusion. Propofol infusion started at a predicted plasma propofol concentration of 2 μg/ml for 10 min to allow time for elimination of isoflurane from the animal before the experiment began. For Feline C, the intravenous catheter was placed while the feline was fully awake and no isoflurane was administered. Eye blink, ear twitch, and toe pinch withdrawal reflexes were examined at each level of propofol infusion.

### Analysis

For AP and LFP analysis, 30-s windows of data were examined during an awake (Feline A, B, and C) or baseline (Patient A and B) state, a lightly anesthetized state (Patient A and B, Feline A, B, and C), a deeply anesthetized state (Patient A and B, Feline A, B, and C), and an isoelectric state (Feline C). The human patient data were all collected intraoperatively under a level of anesthesia appropriate for patient safety and comfort. Therefore, for these data we will be referring to “lightly” and “deeply” anesthetized states only in a relative sense and only to simplify presentation. For the experiments in which propofol was delivered by bolus injection, clinical indicators of depth of anesthesia were not assessed as even the “light” state represents a clinical state consistent with general anesthesia. However, low-pass filtered (200 Hz) voltage traces can be used to categorize the 30-s time windows as belonging to the lightly or deeply anesthetized states by the resemblance of the voltage traces to the EEG waveforms from other human studies during their lightly and deeply anesthetized states, respectively (Brown et al., 2010). A low-pass filter of 200 Hz was applied to the voltage traces to closely approximate EEG waveforms seen in other human anesthesia studies.

The analyses of AP firing rate and LFP multi-taper spectra were performed using the Chronux package (http://chronux.org) (Mitra and Bokil, 2008). For LFPs, low-frequency bands are orders of magnitude larger in power than high-frequency bands (Miller et al., 2009). Therefore, each spectrogram was normalized by frequency bin to enhance visualization across the broad range of frequencies. Each time-frequency point was normalized by the minimum and maximum value of that frequency bin. LFP power in the high gamma band (60–120 Hz) was quantified over 30-s windows in three distinct brain states. The LFP power in the awake (felines) or minimally anesthetized (human patients) state was considered baseline, and the mean percent change from the LFP power in the awake state was calculated across all channels for each brain state. The 30-s periods of data were low-pass filtered at 200 Hz for each subject under varying levels of anesthesia. AP firing rate histogram was smoothed using a Gaussian kernel of 1-s and standard error bars were calculated using a bootstrapping procedure.

The temporal relationship of AP firing and LFP was examined by generating averaged AP-aligned LFP for three different levels of anesthesia. The time periods taken for the AP-aligned LFP analyses started at the same time as the 30-s windows for the awake or baseline state, lightly anesthetized state, and deeply anesthetized state. These time windows taken for analysis were extended by 3 min or longer to obtain enough APs. The temporal relationship between APs and the minimum value of the LFP during a 500-ms epoch around each AP was determined. A 500-ms epoch was chosen to capture the slowest AP-aligned LFP waveforms observed. Using epoch sizes of 200- and 1000-ms produced similar results because of the increased probability an AP would fire very close in time to the LFP minimum. The AP to minimum LFP value was created for each AP on all electrodes for the three different levels of anesthesia. A probability density function was created plotting the difference between the time the minimum value occurred in each LFP epoch and the time the AP fired. The integral of the probability density for the 50-ms around the AP firing function yielded the probability that the minimum value of the LFP and AP firing occurred with 50 ms of each other, i.e., they had a consistent temporal relationship. Randomly generated AP times were used to serve as a control in these analyses (Destexhe et al., 1999). The number of randomly generated APs was the same as observed in the original data to maintain AP firing rate. Randomly generated AP times were also used to generate averaged LFP epochs and served as a control for this analysis.

## Results

All subjects, both human and feline, tolerated the experiments well. There were no adverse events relating to the propofol administrations. The human subjects enjoyed a routine postoperative course. Feline subjects were in good condition post-experiment.

**Figure 1:**
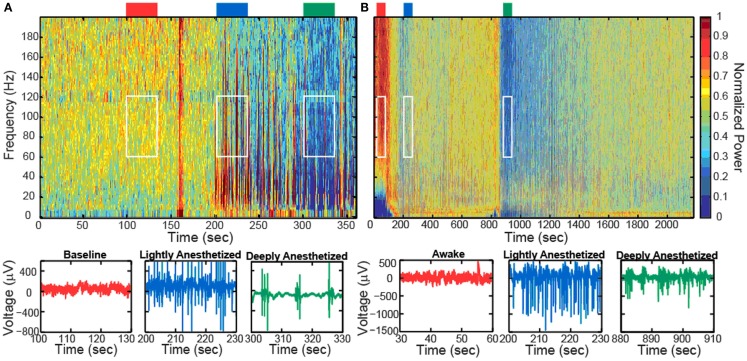
**Boluses of propofol decreased the high-frequency power in the LFP spectra spectrogram and low-pass filtered traces (200 Hz) from one representative electrode for Patient A (A) and Feline A (B)**. Patient A received one bolus of at ∼200 s, while Feline A received two boluses of propofol at ∼100 and ∼850 s. Colored rectangles above the spectrograms correspond with the level of anesthesia. Red represents the baseline or awake state. Blue represents the lightly anesthetized state. Green represents the deeply anesthetized state. White rectangles indicate the time periods and frequency bands chosen for the average power calculations across the array.

**Figure 2:**
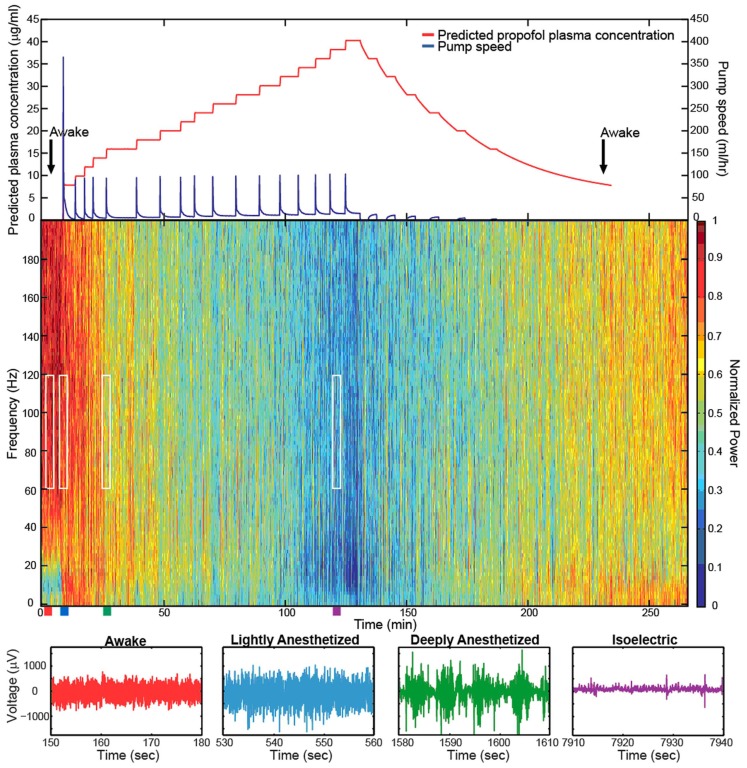
**Target-controlled infusion of propofol gradually decreased the high-frequency power in the LFP spectra**. Spectrogram and low-pass filtered traces (200 Hz) from one representative electrode in Feline C. Top plot show the predicted propofol plasma concentration using the target-controlled infusion system (red) and the TCI pump speed (blue) over the duration of the experiment. Induction and emergence from anesthesia occurred at nearly the same propofol concentrations as noted by the black arrows. Colored rectangles below the spectrogram correspond with the level of anesthesia. Red represents the awake state. Blue represents the lightly anesthetized state. Green represents the deeply anesthetized state. Purple represents the isoelectric state. White rectangles indicate the time periods and frequency bands chosen for the average power calculations across the array. With very high propofol concentrations, power in the LFP decreased across all frequency bands. After the peak propofol concentration is reached and the propofol concentration decreases, the power in the LFP increases.

Increased propofol concentration resulted in suppression of small-amplitude, high-frequency activity, and a dominance of large-amplitude, low-frequency spike-like activity in the LFP. For both patients, the LFP was initially a small-amplitude, high-frequency oscillatory signal in the baseline period (BIS = ∼50). As the anesthesia was increased, the LFP developed into a larger-amplitude spike-like signal. In the deeply anesthetized state, the LFP consisted of large-amplitude spike-like activity separated by silent periods, which resemble EEG “bursts” (Figure 1A; Table 1). We wished to evaluate whether the changes in LFP power seen in the human subjects could also be observed in an animal model under more controlled conditions, i.e., more disparate brain states and during steady states of anesthesia. In Feline A, continuous recordings were made from a fully awake state and two separate boluses of propofol were administered. During this feline model experiment, the observed changes in LFPs were similar to those observed in the human subjects (Table 1). To provide a controlled, stepwise administration of propofol, a pharmacokinetic model was developed using a TCI system for Felines B and C. During the controlled infusion of propofol, similar patterns of change in LFP power to those observed during bolus injections were observed. For Felines B and C, the LFP power in the high frequencies gradually decreased as the predicted propofol plasma concentration increased (Table 1). Additionally, the emergence from propofol hypnosis can be observed in the recordings as a return of power in the higher frequencies several hundred seconds after bolus injection (Figure 1B) and as the target concentration of propofol was reduced (Figure 2).

**Table 1:** **Percent change in average power of a 30-s time window in the high gamma band (60–120 Hz) with respect to the baseline or awake state for all electrodes (*N* = 96; mean ± SD)**.

Subject	Lightly anesthetized (L; %)	Deeply anesthetized (D; %)	Pairwise significance
Patient A	16.01 ± 13.48	−28.67 ± 7.6	All pairs
Patient B	−4.96 ± 11.86	−10.09 ± 10.39	All pairs
Feline A	−29.79 ± 5.69	−41.35 ± 7.31	All pairs
Feline B	−20.22 ± 6.57	−22.69 ± 6.66	A/B-L, A/B-D
Feline C	−7.23 ± 3.38	−23.04 ± 2.84	All pairs

*All changes in power were significant (Kruskal–Wallis, *p* < 0.01; multiple comparisons test, *p* < 0.05; A/B, awake/baseline; L, lightly anesthetized; D, deeply anesthetized)*.

The level of propofol correlated with the level of consciousness in both Felines B and C. Feline B in the lightly anesthetized state (predicted plasma propofol concentration = 6 μg/ml) made small voluntary movements and responded to touch. Eye blink, ear twitch, and toe pinch reflexes were still present at this level of anesthesia. In the deeply anesthetized state (predicted plasma propofol concentration = 8 μg/ml), only the eye blink reflex was present. For Feline B, an isoelectric state was not reached at the maximum level of anesthesia tested (predicted plasma propofol concentration of 18 μg/ml). Feline C in the lightly anesthetized state (predicted plasma propofol concentration = 8 μg/ml) made small voluntary movements and responded to touch. Eye blink, ear twitch, and toe pinch reflexes were still present at this level of anesthesia. In the deeply anesthetized state (predicted plasma propofol concentration = 14 μg/ml), the LFP developed bursts of large-amplitude LFP separated by silent periods, i.e., burst suppression. Voluntary movement stopped, but eye blink, ear twitch, and toe pinch reflexes were present at this level of anesthesia. As anesthesia was increased only the eye blink reflex remained at a predicted plasma propofol concentration of 18 μg/ml, and all reflexes were absent at a predicted plasma propofol concentration of 30 μg/ml. LFP power returned in the higher frequencies as the predicted plasma propofol concentration was decreased from 40 μg/ml (Figure 2).

**Figure 3:**
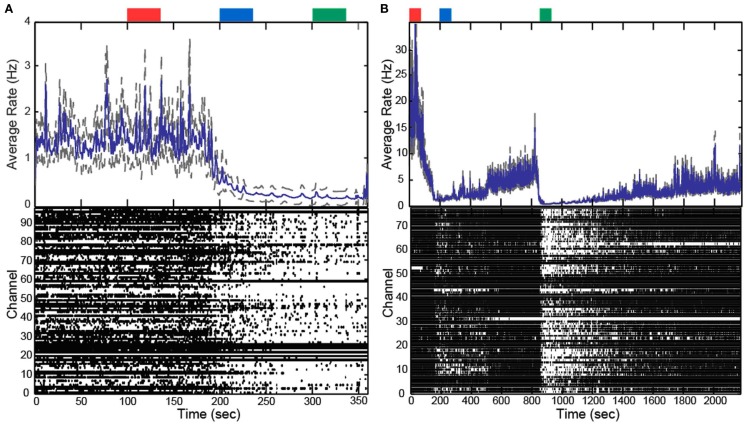
**Boluses of propofol decreased firing rate across array**. Raster plots and firing rate of Patient A **(A)** and Feline A **(B)**. Patient A received one bolus of at ∼200 s, while Feline A received two boluses of propofol at ∼100 and ∼850 s. Propofol boluses resulted in decreased AP firing rate as seen in the raster plots and firing rate histograms. The blue line represents the firing rate histogram and the gray dashed lines represent an interval of 4 standard errors wide centered at the mean. Colored rectangles above the plots correspond with time periods chosen for the average firing rate calculations. Red represents the baseline or awake state. Blue represents the lightly anesthetized state. Green represents the deeply anesthetized state. With the emergence from anesthesia, the firing rate begins to increase in Feline A after both boluses of propofol.

**Figure 4:**
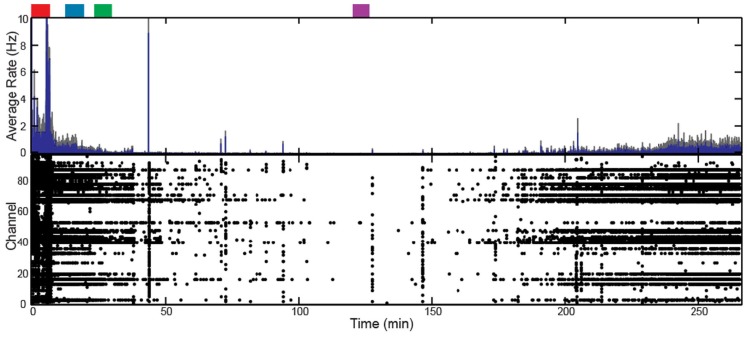
**Target-controlled infusion of propofol decreased firing rate across the array for Feline C**. Controlled infusion of propofol resulted in decreased AP firing rate as seen in the raster plot and firing rate histogram. The blue line represents the firing rate histogram and the gray dashed lines represent an interval of 4 standard errors wide centered at the mean. Colored rectangles above the plots correspond with time periods chosen for the average firing rate calculations. Red represents the awake state. Blue represents the lightly anesthetized state. Green represents the deeply anesthetized state. Purple represents the isoelectric state. With the emergence from anesthesia, the firing rate begins to increase 175 min into the recording.

In addition to altering the spectral power of LFPs, propofol administration also resulted in decreased AP firing rate. For Patients A and B, the average AP firing rate generally decreased across all electrodes in the array when comparing the baseline state with the lightly and deeply anesthetized states. For Feline A, the average AP firing rate decreased across all electrodes in the array comparing the awake state with the lightly and deeply anesthetized states. The recovery from the propofol boluses can be observed in the recording as return to higher AP firing rates over several hundred seconds after each bolus (Figure 3). The controlled infusion of propofol also yielded changes in AP firing rate similar to those observed during bolus injections. For Felines B and C, the average AP firing rates across the array gradually decreased as the predicted propofol plasma concentration increased (Table 2). For Feline C, APs in the isoelectric state were rare (predicted plasma propofol concentration = 40 μg/ml), but AP firing rates returned to higher levels as the propofol concentration was decreased (Figure 4).

**Table 2:** **Average firing rate for a 30-s time window for all subjects across all electrodes (*N* = 96; mean ± SD)**.

	Awake/baseline (A/B; Hz)	Lightly anesthetized (L; Hz)	Deeply anesthetized (D; Hz)	Pairwise significance
Patient A	1.426 ± 1.912	0.4139 ± 0.9779	0.1772 ± 1.121	All pairs
Patient B	0.8781 ± 0.9563	0.9014 ± 0.9805	0.2208 ± 0.3181	A/B-DL-D
Feline A	15.77 ± 16.62	0.8125 ± 0.8182	0.1663 ± 0.2454	All pairs
Feline B	0.6538 ± 2.107	0.0705 ± 0.4123	0.0687 ± 0.4518	A/B-L, A/B-D
Feline C	0.3993 ± 0.9782	0.150 ± 0.3644	0.0257 ± 0.0821	A/B-D

*Values are mean ± SD*.

*All changes in firing rate were significant (Kruskal–Wallis, *p* < 0.01; multiple comparisons test, *p* < 0.05; A/B, awake/baseline; L, lightly anesthetized; D, deeply anesthetized)*.

**Figure 5:**
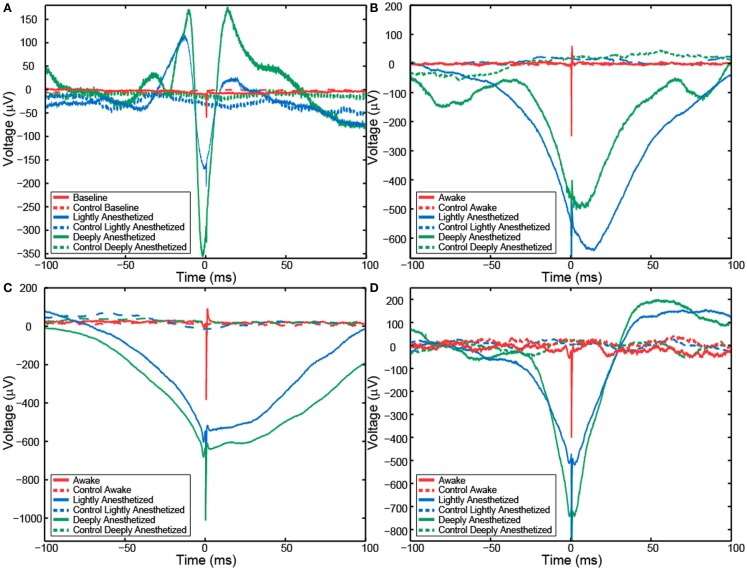
**Action potential-aligned LFP plots for one representative channel from Patient A (A), Feline A (B), Feline B (C), and Feline C (D) in three distinct brain states are shown**. AP-aligned LFP exhibited a negative-going spike-like potential proximal in time to the APs. As the anesthesia increased, the amplitude of the spike-like LFP increased. Dashed lines represent the control cases in which randomly generate AP times were used to align the LFP. Red represents the awake state. Blue represents the lightly anesthetized state. Green represents the deeply anesthetized state.

Although AP firing rates and LFP activity were altered by anesthesia, AP-aligned LFP analysis demonstrated the temporal relationship between them remained relatively consistent across the different levels of anesthesia. AP-aligned LFP exhibited a negative-going potential proximal in time to the APs. As the anesthesia increased, the AP-aligned LFP exhibited large-amplitude spike-like activity (Table 3). Because the number of well-isolated APs for Patient B was small, the AP-aligned LFP data from this subject were not analyzed. The phenomenon was also observed during the controlled infusion experiments. The LFP and AP temporal relationship was relatively consistent across all levels of anesthesia, i.e., APs fired during a local minimum in the LFP (Figure 5).

**Table 3:** **The average minimum across all electrodes (*N* = 96) for the AP-aligned LFP analysis (mean ± SD)**.

	Baseline/awake/baseline (A/B; μV)	Lightly anesthetized (L; μV)	Deeply anesthetized (D; μV)	Pairwise significance
Patient A	−23.16 ± 14.46	−217.22 μV ± 150.18	−204.96 ± 190.24	A/B-L, A/B-D
Feline A	−47.28 ± 22.75	−354.90 ± 155.38	−280.02 ± 148.7	A/B-L, A/B-D
Feline B	−133.36 ± 47.65	−766.16 ± 312.72	−615.28 ± 285.72	A/B-L, A/B-D
Feline C	−141.93 ± 56.83	−582.29 ± 290.61	−577.45 ± 323.40	A/B-L, A/B-D

*All changes in power were significant (Kruskal–Wallis, *p* < 0.01; multiple comparisons test, *p* < 0.05; A/B, awake/baseline; L, lightly anesthetized; D, deeply anesthetized)*.

**Figure 6:**
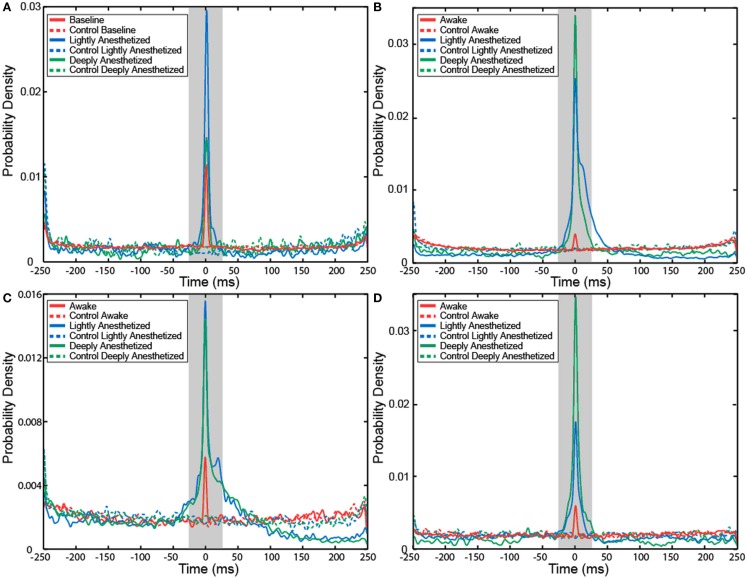
**Probability density of the temporal separation between APs and the minimum LFP value for each AP on all electrodes for Patient A (A), Feline A (B), Feline B (C), and Feline C (D) at three different levels of anesthesia**. The integral of the probability density for the 50-ms around AP firing (shaded region) yielded the probability that the minimum value of the LFP and AP firing occurred with 50 ms of each other, i.e., they had a consistent temporal relationship. With propofol administration, the probability of an AP occurring at the local minimum of the LFP increased. Dashed lines represent the control cases in which randomly generated AP times were used to align the LFP. Red represents the awake or baseline state. Blue represents the lightly anesthetized state. Green represents the deeply anesthetized state.

The probability that an AP would fire when the LFP reaches this local minimum increased with propofol administration. A probability density function was created plotting the difference between the time the minimum value occurred in each LFP epoch and the time the AP fired. The integral of the probability density for the 50-ms around the AP firing function yielded the probability that the minimum value of the LFP and AP firing occurred with 50 ms of each other, i.e., they had a relatively consistent temporal relationship compared to control. Randomly generated AP times were used to generate averaged LFP epochs and served as a control for this analysis. For each subject, the probability that the minimum value of the LFP and AP firing had a consistent temporal relationship increased in the lightly and deeply anesthetized states from baseline (Table 4; Figure 6).

**Table 4:** **Probability that minimum value of the LFP would occur within 50 ms of an AP for all electrodes**.

	Awake/baseline	Lightly anesthetized	Deeply anesthetized
Patient A	0.15 (0.09)	0.31 (0.06)	0.19 (0.08)
Feline A	0.10 (0.08)	0.48 (0.08)	0.40 (0.09)
Feline B	0.11 (0.08)	0.30 (0.09)	0.27 (0.09)
Feline C	0.11 (0.09)	0.27 (0.09)	0.45 (0.09)

*Values in parentheses refer to control probabilities gene*.

## Discussion

Previous studies of propofol in humans have used macroscopic EEG and ECoG electrodes, which integrate neural signals from large areas of the brain and cannot record APs from individual neurons. In the current study, UEAs were used to examine changes in high-frequency micro-scale LFPs and in APs due to propofol administration in the human and feline. As hypothesized, increased propofol concentration decreased the high gamma (60–120 Hz) power in the LFP spectra and decreased AP firing rates in the neocortex. The temporal relationship between APs and LFPs remained relatively consistent across all levels of anesthesia, while the probability that an AP would fire when the LFP reach this local minimum increased from baseline with propofol administration. The changes in neural activity were correlated with decreased responsiveness, i.e., the level of consciousness.

In non-human primates, APs and LFPs were phase-locked in V4 during attention to visual stimuli (Fries et al., 2001), parietal cortex during activation of working memory (Pesaran et al., 2002), and motor cortex during voluntary movements (Donoghue et al., 1998). In human patients, APs and LFPs were observed to be phase-locked in the hippocampus, superior temporal gyrus, entorhinal cortex, orbitofrontal cortex, and amygdala (Jacobs et al., 2007). These non-human primate and human studies support that neurons represent information in terms of the timing of APs relative to neuronal oscillations.

We observed in the anesthetized states regularly occurring large-amplitude, spike-like potentials, and burst suppression patterns replaced the small-amplitude, high-frequency oscillatory LFPs seen in the awake state, and AP firing was concomitantly decreased. However, the AP–LFP temporal relationship was maintained and the probability of an AP firing at the LFP local minimum was increased in the lightly and deeply anesthetized state compared to the awake and baseline states. A clear pattern of increasing probability not consistently seen between the lightly and deeply anesthetized states may be due to moderately high levels of anesthesia in the lightly anesthetized state. These anesthesia-induced changes in AP and LFP structure resulted in a decreased number of distinct neural activity patterns and therefore lowered the information capacity of the neural signal (Shannon and Weaver, 1949; Alkire et al., 2008). The entrainment of APs at low-frequency LFPs induced by propofol anesthesia would impair the amount of information represented in terms of the timing of APs relative to neuronal oscillations and decrease information processing of the cortex. Therefore, the effects of propofol anesthesia may be better characterized as a decrease in cortical information processing rather than as general neuronal inactivation.

That AP firing occurs when the transmembrane potential reaches a threshold voltage is well established (Hodgkin and Katz, 1949); however, the relationship between the LFP and the transmembrane potential is less well understood. The transmembrane potential is the integration of the excitatory and inhibitory inputs onto a particular neuron, while the LFP represents the integration of the post-synaptic potentials from all neurons within several hundred micrometers of the recording electrode, i.e., the integration of the excitatory and inhibitory inputs to the local cortical circuitry. We did not observe that the probability of an AP occurring was related to a voltage threshold of the LFP; however, we observed that AP firing occurred with a high probability at a local minimum of the LFP. Across all levels of consciousness, AP firing was temporally correlated with the structure of the LFP, e.g., inflection points or minima, rather than a specific voltage level of the LFP. The occurrence of an inflection point in the LFP may reflect the moment that a significant shift occurs in the relative excitatory and inhibitory balance of the local network. GABA_A_ inhibition induced by propofol alters the balance between excitatory and inhibitory activity in cortical circuits, and this altered balance is likely related to the observed changes in the LFP and the AP–LFP relationship. Simultaneous intracellular and extracellular recordings in awake rats using AP-aligned LFP demonstrated that the LFP is correlated with the transmembrane depolarization of a single neuron (Okun et al., 2010). This finding, together with the consistently observed LFP–AP temporal relationship observed in the current study, suggest that there may be a biophysical mechanistic linkage between LFPs and APs. Many important neurophysiological animal studies are performed under various levels of hypnosis and anesthesia. Using intraperitoneal thiopental sodium to induce a lightly anesthetized state, and reduce AP firing due to top-down feedback and other processes, allowed the examination of AP firing in V1 driven by feedforward signals evoked by visual stimuli (Hubel and Wiesel, 1959). Using a stimulus presentation paradigm that only allowed top-down feedback LFP signals to be recorded by electrodes in V1, it was possible to classify natural images based on decoding of the LFPs at above chance levels in the awake monkey, but only at chance levels in the anesthetized monkey (Shushruth et al., 2011). These studies show that varying the level of anesthesia can differentiate feedforward and feedback signals in V1. Similarly, studies examining AP–LFP relationships in V1 using AP-aligned LFP analysis achieved differing results under different levels of anesthesia. One study used awake monkeys (Ray and Maunsell, 2011) and another used anesthetized monkeys and felines (Nauhaus et al., 2009). Intracortically recorded LFPs are thought to be generated by synaptic potentials (Mitzdorf, 1985; Nunez and Srinivasan, 2006; Katzner et al., 2009; Khawaja et al., 2009) that arise from multiple neural sources, and anesthesia can differentially alter the balance between these sources. Propofol binds to GABA_A_ receptors that are located primarily in the cortex, so that propofol may impact corticocortical inputs more strongly than subcortical inputs. These studies and the present work demonstrate that while anesthetized preparations provide control of experimental paradigms and are a powerful tool to dissect neurophysiological processes, the impact of the anesthesia itself on the neural system being studied must be taken into account when interpreting the results.

On the administration of propofol we consistently observed a decrease in high-frequency power and AP firing rate, and an increase in regular and predictable patterns of LFP and AP activity. This reduced the information processing capacity in the neocortex and was correlated with a loss of responsiveness and consciousness. These effects of propofol on APs and LFPs have important implications for the processing of information in the neocortex, and therefore on the interpretation of experimental results in anesthetized preparations.

## Author Contributions

Paul House, Bradley Greger, and Talmage D. Egan conception and design of research; Paul House, Bradley Greger, Sara J. Hanrahan, Rebecca A. Parker, Takahiro Ogura, and Shinju Obara performed experiments; Sara J. Hanrahan and Bradley Greger analyzed data; Bradley Greger, Paul House, and Talmage D. Egan interpreted results of experiments; Sara J. Hanrahan prepared figures; Sara J. Hanrahan and Bradley Greger drafted manuscript; Bradley Greger, Paul House, and Talmage D. Egan edited and approved final version of manuscript.

## Conflict of Interest Statement

The authors declare that the research was conducted in the absence of any commercial or financial relationships that could be construed as a potential conflict of interest.
